# Perinatal and Other Risk Factors for Common Infections in Infancy: A Prospective Cohort Study

**DOI:** 10.1097/INF.0000000000004112

**Published:** 2023-09-14

**Authors:** Sanni Hyvönen, Terhi Tapiainen, Tytti Pokka, Terhi Solasaari, Katri Korpela, Willem M. de Vos, Anne Salonen, Kaija-Leena Kolho

**Affiliations:** From the * Department of Pediatrics, Tampere University Hospital, Tampere, Finland; † Department of Pediatrics, Faculty of Medicine, University of Helsinki, Helsinki, Finland; ‡ Research Unit of Clinical Medicine and Medical Research Centre Oulu, University of Oulu, Oulu, Finland; § Department of Pediatrics and Adolescent Medicine, Oulu University Hospital, Oulu, Finland; ¶ Biocenter Oulu, University of Oulu, Oulu, Finland; ∥ Research Service Unit, Oulu University Hospital, Finland; ** Pediatric Clinic, Social Services and Health Care Division, City of Helsinki, Helsinki, Finland; †† Human Microbiome Research Program, Faculty of Medicine, University of Helsinki, Helsinki, Finland; ‡‡ Laboratory of Microbiology, Wageningen University, the Netherlands; §§ Department of Pediatric Gastroenterology, Children’s Hospital, Helsinki University, Helsinki, Finland; ¶¶ Department of Pediatrics, Faculty of Medicine and Health Technology, University of Tampere, Tampere, Finland.

**Keywords:** birth cohort, children, infectious diseases, newborn, respiratory

## Abstract

**Objective:**

Limited data from prospective cohort studies in high-income countries are available on the perinatal risk factors for common infections in children. Our hypothesis was that perinatal factors may be risk factors for infectious episodes during the first year of life.

**Methods:**

In this prospective Health and Early Life Microbiota birth cohort study of full-term infants (n = 1052) born in 2016–2018, the number and duration of infection episodes were collected online at weekly to monthly intervals. In a multivariate regression model, the main exposures were perinatal factors such as mode of delivery and intrapartum antibiotics. Environmental factors were additional exposures. The outcomes were the number and duration of infectious episodes in the first year of life.

**Results:**

The mean number of infection episodes was 4.2 (2.9 SD). The mean duration of infection symptoms was 44 days (40 SD). Upper respiratory infections accounted for 83% of the episodes (3674/4455). Perinatal factors were not associated with the number nor the duration of infection episodes, but cesarean section was associated with an increased occurrence of urinary tract infections in infancy [adjusted odds ratio (aOR): 3.6; 95% confidence interval (CI): 1.13–11.1]. Of the additional exposures male sex (aOR: 1.1; 95% CI: 1.0–1.2) and the presence of siblings (aOR: 1.3; 95% CI: 1.2–1.4) were associated with the number of infection episodes.

**Conclusions:**

This prospective cohort study showed that perinatal factors, mode of delivery and intrapartum antibiotics were not associated with the risk of common infections in infancy, but cesarean delivery was associated with a risk of urinary tract infections.

Respiratory tract infections (RTIs) and gastrointestinal infections (GIs) are common in children,^1–4^ causing a burden on health care and the economy and reducing the quality of life.^5,6^ Healthy children have a variable frequency of common infections,^2,4,5^ in which previously reported known epidemiological risk factors include male sex,^7,8^ day care,^3,4,9–11^ the presence of siblings,^2,4,10,12^ parental smoking^13^ and the duration of breastfeeding.^14^

Although the risks of RTIs and lung health,^15,16^ GIs,^17^ urinary tract infections (UTIs)^18^ and infections during chemotherapy^19^ in children have been shown to be associated with the composition of the gut and respiratory microbiota, limited data are available on the perinatal factors previously shown to modify gut and respiratory tract colonization^20,21^ as risk factors for common infections in childhood in prospective cohort studies in high-income countries.^1–4,10^

Given the hypothesis that perinatal factors may be predictive of the number and duration of infection episodes in infancy, we set out in this prospective cohort study to investigate in a cohort of term infants the perinatal and other risk factors for common infections during the first year of life.

## METHODS

### Study Design

**FIGURE 1.**
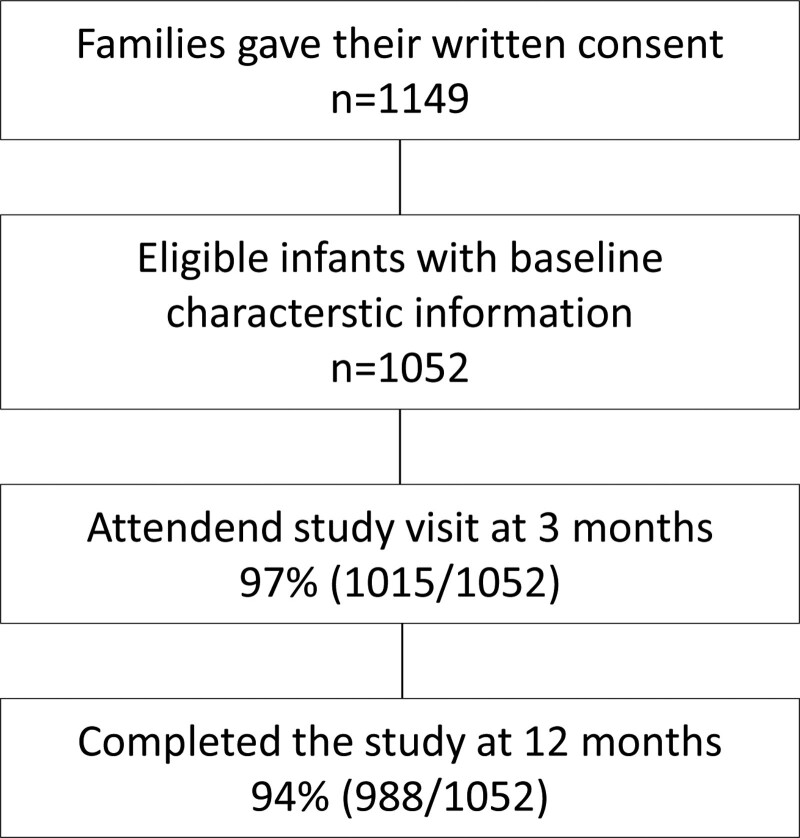
Flow chart.

HELMi (Health and Early Life Microbiota) is a longitudinal, prospective general population birth cohort study, set up to identify environmental, lifestyle and genetic factors that modify the development of the intestinal microbiota in the first years of life and their relation to child health.^22^ Of 1149 families giving written consent, we included 1052 healthy singleton infants born on gestational weeks 37–42 without any known congenital defects, for whom complete information on childbirth, sex and family background were recorded (Fig. 1). In brief, the families, living mainly in the Helsinki region of Finland, were recruited between February 2016 and March 2018, that is, before the COVID-19 pandemic. The data were collected prospectively at weekly to monthly intervals, and the retention rate at 12 months was 94% (n = 988/1052). The proportion of highly educated mothers (80%) in the group that did not complete the questionnaires and diaries for the whole first year of life (n = 64), was statistically significantly lower than in the total cohort (*P* = 0.03). Otherwise, the responding and nonresponding groups did not show any significant differences in baseline characteristics.

We have reported baseline characteristics of the HELMi cohort earlier.^22^ Briefly, the proportion of infants born via cesarean section was 17% (Table 1), reflecting the general proportion of cesarean section births in Finland (16.7% in 2017).^23^ In addition to previously reported characteristics, the children were exclusively breastfed up to a mean age of 3.3 months (1.8 SD) and partially breastfed up to 10 months (3.3 SD). By 12 months of age 77% of the children were fully vaccinated against rotavirus, pneumococcus, diphtheria–tetanus–pertussis–polio–Hib, measles–mumps–rubella and varicella-zoster virus in accordance with the national immunization program. Altogether 4.8% of the children started day care in the first year of life. In addition to the regular electronic online data collection from the families, 97% (n = 1015/1052) of the infants attended a health status examination at the age of 3 months.^22^

**TABLE 1.** Baseline Characteristics of Study Population (n = 1052)

	Categorical: n (%) Numeric: Mean (SD)
Gender, male	532 (51%)
Gestational weeks at birth	40 (1.2 SD)
Birth weight (kg)	3.56 (0.4 SD)
Year of birth	
2016	420 (40%)
2017	570 (54%)
2018	62 (6%)
Season of birth	
Winter	202 (19%)
Spring	241 (23%)
Summer	320 (30%)
Autumn	289 (28%)
Mother’s age	32.8 (4.0 SD)
Father’s age	34.8 (5.2 SD)
Mode of delivery	
Cesarean delivery	176 (17%)
Antibiotics (yes)	176 (100%)
Vaginal birth	876 (83%)
Antibiotics (yes)	208 (20%)
Number of siblings in household	
0 siblings	514 (49%)
1 sibling	416 (40%)
2 siblings	98 (9.3%)
≥3 siblings	24 (2.3%)
Breastfeeding yes	1039 (99%)
Exclusive (months)	3.3 (1.8 SD)
Partial (months)	10.4 (3.3 SD)
Breastfeeding exclusive <4 months	490 (47%)
Maternal education	
Secondary school	8 (0.8%)
Upper secondary/vocational school	117 (11%)
University including polytechnic	927 (88%)
Paternal education	
Secondary school	24 (2.3%)
Upper secondary/vocational school	245 (23%)
University including polytechnic	768 (73%)
Maternal smoking (yes)	7 (0.7%)
Use of probiotics during pregnancy	742 (71%)
At least 1 parent with asthma	134 (13%)
Mother with asthma	92 (8.7%)
Father with asthma	48 (4.6%)
At least 1 parent with an autoimmune disease	162 (15.4%)
Mother with an autoimmune disease	102 (9.7%)
Celiac disease	20 (1.9%)
Inflammatory bowel disease	22 (2.1%)
Thyroid disease	44 (4.2%)
Type 1 diabetes	3 (0.3%)
Psoriasis	7 (0.7%)
Rheumatic disease	6 (0.6%)
Father with an autoimmune disease	65 (6.2%)
Celiac disease	9 (0.9%)
Inflammatory bowel disease	24 (2.3%)
Thyroid disease	4 (0.4%)
Type 1 diabetes	13 (1.2%)
Psoriasis	10 (1.0%)
Rheumatic disease	5 (0.5%)
Started day care in the first year of life	51 (4.9%)
Vaccination	
Full programme	806 (77%)
Partial programme	108 (10%)
None	33 (3%)
Furry pet at home	378 (36%)

Data missing (n): Father’s age (17), Paternal education (15), Started day care in the first year of life (83), vaccination (105).

The study was conducted in accordance with the principles of the Helsinki Declaration and the protocol was reviewed by the ethical committee of the Helsinki and Uusimaa Hospital District (263/13/03/03 2015), Finland. All the families gave their written informed consent in advance.

### Clinical Follow-up

A prospective clinical follow-up was conducted using electronic questionnaires that stored the answers instantly in an online database. The parents filled in the questionnaire form weekly for the first 4 months, every 2 weeks until 7 months and monthly from 8 to 12 months. In addition, they filled in comprehensive questionnaires that included childcare practices, living conditions and infant health at 3-month intervals. The parents received automatic reminders via email and text message to promote compliance with this questionnaire program.^22^

Families recorded daily infection episodes and infection-related symptoms and reported them in a continuous manner using an online study diary. The infections recorded in this way were classified as follows: (1) Respiratory tract infections (RTI): upper (URTI) and lower (LRTI) respiratory tract infections, (2) URTI: common cold, cough, laryngitis, influenza, otitis media, tonsillitis and pharyngitis, (3) LRTI: pneumonia, acute obstructive bronchitis, bronchiolitis, (4) GI: diarrhea, vomiting, (5) UTI: pyelonephritis and any UTI and (6) other infections (see Table, Supplemental Digital Content 1, http://links.lww.com/INF/F264).

### Statistical Analysis

The main exposures of interest were delivery mode and exposure to intrapartum antibiotics. Other exposures and covariates were the number of siblings, breastfeeding, the use of probiotics during pregnancy, parental asthma, maternal education status, day-care attendance and presence of furry pets in the household. The outcomes were the number of infection episodes and the number of infection-related symptom days during the first year of life. For the statistical analysis, an infection episode was defined by the first and last dates with symptoms that the families reported. If the dates of different symptoms overlapped or started on the day after another symptom, they were considered to form 1 episode. The infections were categorized according to the symptoms.

We used a multivariate Poisson regression model to estimate the association between exposures and outcomes. Means, SDs, medians and interquartile ranges (IQR) were reported for the infections. The multivariate analyses included the following variables: sex, year and season of birth, mode of delivery, intrapartum antibiotics, number of siblings, parental asthma, maternal education, use of probiotics during pregnancy and presence of furry pets in the household. Maternal education and the presence of siblings were grouped into 2 category variables in the analyses. The analyses were performed with SPSS version 28, and the results were expressed as adjusted odds ratios with 95% confidence intervals (95% CIs). We estimated the required sample size for the observational cohort study using StatsDirect Statistical Software. We regarded OR of 1.25 as a clinically significant risk for both cesarean section and exposure to intrapartum antibiotics during vaginal birth. We chose statistical power of 80% and alpha error of 5%. The required sample size, with a ratio of 1:4 for exposed and unexposed subjects, was 165 exposed and 660 unexposed participants.

## RESULTS

### Infections in the Cohort

The 1052 full-term infants who met the inclusion criteria (Fig. 1) and were followed up with online data collection for the first year of life had a total of 4455 infection episodes, affecting 90% of them (Table 2). The mean numbers of infections per child for the year and for each 3-month period can be seen in Table 2. The mean age at the first infection episode was 3.4 months (SD 2.8; median 3.0 months; IQR: 1.0–5.0). Children had mostly RTIs (3688 episodes) during the first year of life, and URTIs alone accounted for 83% (3674 episodes) of all the episodes, followed by other infections 12% (515 episodes), GIs 5.4% (239 episodes), LRTIs 0.3% (14 episodes) and UTIs 0.3% (13 episodes) (Table 2).

**TABLE 2.** Incidence and Duration of Infectious Episodes in 1052 Full-term Children During Their First Year of Life

			Frequency of Infections Per Child		No. of Days With Infection Per Child	
	Total Number of Episodes	Proportion of Children With Episodes (No.)	Mean (SD)	Median (IQR)	Mean (SD)	Median (IQR)
All infections						
0–12 months	4455	90% (944)	4.2 (2.9)	4.0 (2.0–6.0)	44 (40)	35 (15–63)
0–3 months	916	54% (570)	0.9 (1.0)	1.0 (0.0–1.0)	9 (14)	3 (0.0–13)
4–6 months	1119	63% (663)	1.1 (1.0)	1.0 (0.0–2.0)	11 (15)	7 (0.0–16)
7–9 months	1143	63% (660)	1.1 (1.1)	1.0 (0.0–2.0)	12 (15)	7 (0.0–17)
10–12 months	1277	65% (684)	1.2 (1.2)	1.0 (0.0–2.0)	13 (17)	8 (0.0–19)
RTI						
0–12 months	3688	89% (913)	3.5 (2.6)	3.0 (1.0–5.0)	41 (39)	31 (12–59)
0–3 months	810	50% (520)	0.8 (1.0)	0.0 (0.0–1.0)	8 (13)	0 (0.0–13)
4–6 months	969	58% (613)	0.9 (1.0)	1.0 (0.0–2.0)	11 (14)	6 (0.0–15)
7–9 months	936	55% (582)	0.9 (1.0)	1.0 (0.0–2.0)	11 (15)	5 (0.0–16)
10–12 months	973	56% (592)	0.9 (1.0)	1.0 (0.0–2.0)	12 (16)	6 (0.0–17)
URTI						
0–12 months	3674	87% (913)	3.5 (2.6)	3.0 (1.0–5.0)	41 (38)	31 (12–59)
0–3 months	806	50% (520)	0.8 (1.0)	0.0 (0.0–1.0)	8 (13)	0 (0.0–13)
4–6 months	965	58% (611)	0.9 (1.0)	1.0 (0.0–2.0)	10 (14)	6 (0.0–15)
7–9 months	935	55% (582)	0.8 (1.0)	1.0 (0.0–2.0)	11 (15)	5 (0.0–16)
10–12 months	968	56% (591)	0.9 (1.0)	1.0 (0.0–2.0)	12 (16)	6 (0.0–17)
LRTI						
0–12 months	14	1.1% (12)	0.01 (0.1)	0 (0.0–0.0)	0.3 (4)	0 (0.0–0.0)
0–3 months	4	0.4% (4)	0.004 (0.6)	0 (0.0–0.0)	0.03 (0.6)	0 (0.0–0.0)
4–6 months	4	0.4% (4)	0.004 (0.06)	0 (0.0–0.0)	0.1 (3)	0 (0.0–0.0)
7–9 months	1	0.1% (1)	0.001 (0.03)	0 (0.0–0.0)	0.01 (0.3)	0 (0.0–0.0)
10–12 months	5	0.5% (5)	0.01 (0.07)	0 (0.0–0.0)	0.1 (2)	0 (0.0–0.0)
UTI						
0–12 months	13	1.0% (10)	0.01 (0.14)	0 (0.0–0.0)	0.1 (1.2)	0 (0.0–0.0)
0–3 months	3	0.3% (3)	0.003 (0.05)	0 (0.0–0.0)	0.01 (0.2)	0 (0.0–0.0)
4–6 months	5	0.5% (5)	0.005 (0.7)	0 (0.0–0.0)	0.05 (0.8)	0 (0.0–0.0)
7–9 months	2	0.2% (2)	0.002 (0.05)	0 (0.0–0.0)	0.02 (0.6)	0 (0.0–0.0)
10–12 months	3	0.1% (1)	0.003 (0.1)	0 (0.0–0.0)	0.02 (0.6)	0 (0.0–0.0)
GI						
0–12 months	239	20% (212)	0.2 (0.5)	0 (0.0–0.0)	1 (4)	0 (0.0–0.0)
0–3 months	22	2% (22)	0.02 (0.1)	0 (0.0–0.0)	0.1 (1.2)	0 (0.0–0.0)
4–6 months	41	6% (41)	0.04 (0.2)	0 (0.0–0.0)	0.2 (1.1)	0 (0.0–0.0)
7–9 months	73	7% (72)	0.07 (0.3)	0 (0.0–0.0)	0.4 (2.3)	0 (0.0–0.0)
10–12 months	103	9% (96)	0.1 (0.3)	0 (0.0–0.0)	0.4 (2)	0 (0.0–0.0)
Other infections						
0–12 months	515	36% (373)	0.5 (0.8)	0 (0.0–1.0)	2.3 (5)	0 (0.0–3.0)
0–3 months	81	7% (81)	0.08 (0.3)	0 (0.0–0.0)	0.5 (3)	0 (0.0–0.0)
4–6 months	104	9% (97)	0.1 (0.3)	0 (0.0–0.0)	0.4 (1.6)	0 (0.0–0.0)
7–9 months	132	11% (119)	0.1 (0.3)	0 (0.0–0.0)	0.5 (1.9)	0 (0.0–0.0)
10–12 months	198	16% (173)	0.2 (0.5)	0 (0.0–0.0)	0.1 (2.7)	0 (0.0–0.0)

GI indicates gastrointestinal infection; LRTI, lower respiratory tract infection; RTI, respiratory tract infection; URTI, upper respiratory tract infection; UTI, urinary tract infection.

Antibiotics were prescribed for 9.2% of the infection episodes, including 10% of URTIs, 71% of the LRTIs, 92% of the UTIs, 0.8% of the GIs and 4.5% of the other infections. Amoxicillin was the most frequently used antimicrobial agent (57%), followed by amoxicillin–clavulanate (18%). The proportion of all infections that were treated in the hospital was 2.4%. Severe infections such as osteomyelitis, cellulitis and sepsis were rare (0.25%) and none of the children died.

### Risk Factors for Infections

#### Perinatal Factors

Multivariate analysis showed no association between the number of overall infection episodes and perinatal factors such as mode of delivery and intrapartum antibiotics (Table 3). The differences in the infection burden between cesarean and vaginal delivery were not statistically significant (95% CI: 0.85–1.01) (Table 3). Children exposed to intrapartum antibiotics had a smaller infection burden (95% CI: 0.88–1.03) than those not exposed to antibiotics, which again were not statistically significant findings (Table 3).

**TABLE 3.** Mode of Delivery and Intrapartum Antibiotics as a Risk Factor for the Occurrence of Infections in Full-term Infants During Their First Year of Life

	Cesarean Delivery n = 176 No. of Episodes Mean (SD), Crude	Vaginal Delivery n = 876 No. of Episodes Mean (SD), Crude	Cesarean Delivery Versus Vaginal Delivery aOR* (95% CI)	Vaginal Delivery, Antibiotics Yes n = 208 No. of Episodes Mean (SD), Crude	Vaginal Delivery, Antibiotics no n = 668 No. of Episodes Mean (SD), Crude	Exposure to Antibiotics in Vaginal Delivery Yes Versus No aOR* (95% CI)
All infections	3.8 (2.5)	4.3 (3.0)	0.93 (0.86–1.01)	4.1 (3.0)	4.4 (3.0)	0.95 (0.88–1.03)
Respiratory tract infections	3.2 (2.4)	3.6 (2.7)	0.93 (0.85–1.02)	3.3 (2.6)	3.7 (2.7)	0.92 (0.83–1.00, *P* = 0.051)
Upper respiratory tract infections	3.2 (2.4)	3.6 (2.7)	0.94 (0.86–1.03)	3.3 (2.6)	3.7 (2.7)	0.92 (0.84–1.00, *P* = 0.050)
Urinary tract infections	0.03 (0.3)	0.01 (0.1)	3.6 (1.1–11.1)	0.01 (0.1)	0.01 (0.1)	1.2 (0.20–5.8)
Gastrointestinal infections	0.2 (0.4)	0.2 (0.5)	0.83 (0.58–1.2)	0.2 (0.5)	0.2 (0.5)	1.0 (0.75–1.4)
Other infections	0.4 (0.7)	0.5 (0.8)	0.90 (0.71–1.2)	0.6 (0.8)	0.5 (0.8)	1.1 (0.92–1.4)

Values are aORs and 95% CIs obtained by using a Poisson regression model.

* All the aORs were adjusted for sex, year of birth, mode of delivery, intrapartum antibiotics, number of siblings, parental asthma, maternal education, use of probiotics during pregnancy and a furry pet in the household.

aOR indicates adjusted odds ratio; CI, confidence interval.

#### Other Factors

Other factors associated with an increase in reported infection symptoms were the use of probiotics during pregnancy (95% CI: 1.1–1.2), male sex (95% CI: 1.0–1.1), birth date in spring (95% CI: 1.0–1.2), the presence of older siblings (95% CI: 1.2–1.4) and the mother’s higher education status (95% CI: 1.1–1.3) (Table 4). Conversely, we found that a furry pet in the household was a protective factor for overall infections (95% CI: 0.9–0.99) (Table 4).

**TABLE 4.** Other Risk Factors for the Occurrence of Infections in Full-term Infants During Their First Year of Life

	All Infections		RTI		UTI	
	No. of Episodes Mean (SD), Crude	*aOR (95% CI)	No. of Episodes Mean (SD), Crude	*aOR (95% CI)	No. of Episodes Mean (SD), Crude	*aOR (95% CI)
Gender						
Girls (n = 520)	4.1 (2.9)	Reference	3.4 (2.6)	Reference	0.01 (0.2)	Reference
Boys (n = 532)	4.4 (2.9)	1.1 (CI: 1.0–1.1)	3.6 (2.7)	1.1 (CI: 1.0–1.2)	0.01 (0.1)	1.2 (CI: 0.4–3.5)
Season of birth						
Summer (n = 320)	4.1 (2.9)	Reference	3.4 (2.7)	Reference	0.01 (0.1)	Reference
Spring (n = 241)	4.5 (2.9)	1.1 (CI: 1.0–1.2)	3.6 (2.6)	1.1 (CI: 0.97–1.2)	0.01 (0.1)	0.7 (CI: 0.1–3.8)
Winter (n = 202)	4.1 (2.9)	1.0 (CI: 0.9–1.1)	3.5 (2.7)	1.0 (CI: 0.9–1.1)	0.02 (0.2)	1.3 (CI: 0.3–5.7)
Autumn (n = 289)	4.2 (2.9)	1.0 (CI: 0.95–1.1)	3.5 (2.7)	1.0 (CI: 0.9–1.1)	0.01 (0.1)	1.0 (CI: 0.3–4.5)
Number of siblings						
0 siblings (n = 514)	3.7 (2.6)	Reference	3.0 (2.3)	Reference	0.01 (0.1)	Reference
≥1 sibling (n = 538)	4.8 (3.1)	1.3 (CI: 1.2–1.4)	4.0 (2.9)	1.3 (CI: 1.2–1.4)	0.01 (0.2)	1.9 (CI: 0.6–5.9)
Parent with asthma						
No (n = 918)	4.3 (2.9)	Reference	3.5 (2.7)	Reference	0.01 (0.1)	Reference
Yes (n = 134)	4.1 (2.8)	0.96 (CI: 0.9–1.1)	3.5 (2.6)	0.97 (CI: 0.9–1.1)	0.01 (0.1)	1.3 (CI: 0.3–5.8)
Parent with an autoimmune disease						
No (n = 890)	4.3 (2.9)	Reference	3.4 (2.7)	Reference	0.01 (0.2)	Reference
Yes (n = 162)	4.1 (3.0)	0.98 (CI: 0.9–1.1)	3.5 (2.6)	1.0 (CI: 0.9–1.1)	0.01 (0.1)	0.95 (CI: 0.2–4.4)
Use of probiotics during pregnancy						
No (n = 310)	3.9 (2.8)	Reference	3.1 (2.5)	Reference	0.02 (0.2)	Reference
Yes (n = 742)	4.4 (3.0)	1.1 (CI: 1.1–1.2)	3.7 (2.7)	1.2 (CI: 1.1–1.3)	0.01 (0.1)	0.3 (CI: 0.1–0.99)
Mother’s education						
Secondary/upper secondary/vocational school (n = 125)	3.7 (2.9)	Reference	3.1 (2.6)	Reference	0.01 (0.1)	Reference
University, including polytechnic (n = 927)	4.3 (2.9)	1.2 (CI: 1.1–1.3)	3.6 (2.7)	1.2 (CI: 1.1–1.3)	0.01 (0.2)	2.2 (CI: 0.3–17.4)
Furry pet at home						
No (n = 674)	4.4 (3.0)	Reference	3.6 (2.7)	Reference	0.01 (0.1)	Reference
Yes (n = 378)	4.0 (2.8)	0.93 (CI: 0.9–0.99, *P* = 0.03)	3.3 (2.5)	0.94 (CI: 0.9–1.01)	0.02 (0.2)	2.3 (CI: 0.8–6.9)
Started day care in the first year of life						
Yes (n = 51)	5.0 (3.2)	—†	4.1 (2.9)	—†	0 (0)	—†
No (n = 918)	4.5 (2.8)		3.7 (2.6)		0.01 (0.2)	
Maternal smoking						
Yes (n = 7)	1.4 (1.8)	—†	0.9 (1.5)	—†	0.4 (1.1)	—†
No (n = 1045)	4.3 (2.9)		3.5 (2.6)		0.01 (0.1)	

Values aORs and 95% CIs are obtained by using Poisson regression model.

* All the aORs were adjusted for sex, year of birth, mode of delivery, intrapartum antibiotics, number of siblings, parental asthma, maternal education, use of probiotics during pregnancy and a furry pet in the household.

† Limited number of observations.

— indicates not applicable; aOR, adjusted odds ratio; CI, confidence interval.

### Risk Factors for Respiratory Infections

#### Perinatal Factors

We found no significant association between the perinatal factors and RTIs (Table 3). Those born via cesarean delivery had fewer RTI episodes (95% CI: 0.85–1.02) and infection days (95% CI: −7.9 to 4.5), figures which were not statistically significant (Table 3). Children exposed to intrapartum antibiotics showed no difference in infection episodes (95% CI: 0.83–1.0) or infection days (95% CI: −9.4 to 2.5) relative to those not exposed (Table 3).

#### Other Factors

The following factors were statistically significantly associated with an increase in reported RTIs episodes: use of probiotics during pregnancy (95% CI: 1.1–1.3), male sex (95% CI: 1.0–1.2), the presence of siblings (95% CI: 1.2–1.4) and the mother’s higher education (95% CI: 1.1–1.3) (Table 4). We found no protective factors. The statistically significant factors for URTIs were the same as for RTIs (Table 4).

### Risk Factors for UTIs

#### Perinatal Factors

UTI was the only infection with a statistically significant association with the mode of delivery, cesarean delivery being associated with an increased mean number of UTIs (95% CI: 1.1–11.1) in the first year of life as compared with those born via vaginal delivery (Table 3). The difference in days of reported UTI-related symptoms was not statistically significant (Table 3). There was no difference in the number of UTI episodes between the infants exposed to intrapartum antibiotics and those not exposed (Table 3).

#### Other Factors

The use of probiotics during pregnancy was a protective factor for UTIs, as it was associated with a decreased mean number of UTI episodes (95% CI: 0.1–0.99) in the first year as compared with children not exposed to probiotics during pregnancy. The consumption of probiotics during pregnancy was not statistically associated with the number of reported UTI symptom days (Table 4).

## DISCUSSION

In this prospective birth cohort study of 1052 infants, perinatal factors, including delivery mode and exposure to intrapartum antibiotics, were not associated with the number of infection episodes during the first year of life or the total number of days with infection symptoms, although the risk of UTIs was associated with the delivery mode, as the infants born via cesarean delivery had an increased risk.

To our knowledge, there are a limited number of prospective birth cohort studies performed in high-income countries that concern perinatal factors, previously shown to modify the colonization and development of the gut and respiratory microbiome,^24,25^ as risk factors for common infections in childhood. Two previous Scandinavian birth cohort studies addressing the effect of cesarean delivery on the risk of childhood infections have reported inconsistent results,^4,26^ while in Denmark, Vissing et al.^4^ found that cesarean delivery increased the risk of LRTIs. A large birth cohort study conducted in Norway was nevertheless unable to find any significant association between cesarean delivery and recurrent LRTIs.^26^ Here, we found no association between perinatal factors (mode of delivery and intrapartum antibiotics) and the occurrence of overall infections or RTIs.

Other factors associated with common infections in childhood have been investigated more extensively. In 5 prospective birth cohort studies from Denmark,^4,10^ Germany^1,3^ and Holland^2^ day-care attendance and the presence of siblings have been reported as risk factors for infections in childhood. The occurrence of infection episodes in the first year of life obtained from our cohort was comparable to those reported in 2 of the above-mentioned birth cohorts studied in Denmark and Germany,^1,4^ whereas the numbers of infections reported in the other cohorts were higher than in the present study.^2,3,10^ However, the differences in the methods of reporting symptoms need to be noted when comparing these results. Only one of the previous birth cohort studies made partial use of electronic data collection,^3^ whereas the present data were collected prospectively with an electronic online system.

UTI has been reported to be one of the leading reasons for intravenous antibiotic treatment for hospitalized children in a high-income country with a comprehensive immunization program.^27^ UTIs have previously been associated with the composition of the gut microbiota,^18^ which in turn has also been associated with perinatal factors such as mode of delivery^24^ and intrapartum antibiotics.^25^ We found no association between intrapartum antibiotics and UTIs, but we did find cesarean delivery to be associated with an increased occurrence of UTIs. This observation supports the idea that there is a link between the maturing gut microbiota and the risk of UTI.

The results of this observational cohort study suggest that the use of probiotics during pregnancy might reduce the occurrence of UTIs in infants. Because of the small sample of UTIs in this cohort, we were not able to assess the effects of different probiotics, the 3 most used being *Lactobacillus rhamnosus*, *Lactobacillus acidophilus* and *Bifidobacterium bifidum*. In any case, the results need to be interpreted with caution because of the high proportion of mothers using probiotics during pregnancy (71%). In particular, the evidence of vertical transfer of prenatal probiotics is inconclusive.^28^

When assessing other associated factors, our results complement previous findings, that male sex is a risk factor for RTIs^2,8,29^ and that the existence of older siblings is negatively associated with RTIs.^2,4,10,12^ We could not verify exclusive breastfeeding as a protective factor, however, although such an association is commonly mentioned in the literature.^14,30–32^ The only protective factor we found was exposure to a furry pet, which was positively associated with the number of overall infections, but not with RTIs as has previously been established.^1,33,34^

The strength of our study lies in the large prospective longitudinal cohort providing comprehensive prospective data on 1052 families. The extensive questionnaires and diaries were collected online at weekly to monthly intervals, and there were few cases of missing data, as most families continued the follow-up until the child reached 1 year of age. As the main focus of the HELMi-birth cohort is gut microbiota development,^20,35^ the data includes over 10,000 biological samples in total (fecal, breastmilk and genetic testing), which can be connected to our study’s infection data in future studies. The strength of the present epidemiological cohort study is that the association of the gut microbiota composition with infectious diseases in infancy in this prospective cohort is underway. This enables the comparison of epidemiological perinatal risk factors presented here and those retrieved from a large-scale bioinformatics analysis regarding gut microbiota composition, and their associations with the occurrence of infectious diseases in infants. We acknowledge that the sample size may be a limitation for some included comparisons. The main limitation of this work is that the cohort represents highly educated mothers in a high-income country. Almost all the mothers breastfed their infants, for instance and very few reported smoking. Thus, the results are not generalizable to child cohorts with different background characteristics. As the number of children starting day care in the first year of life was low, we were not able to assess the impact of day care on the infection burden at this age. It is to be noted that our study shows only associations rather than causality.

## CONCLUSIONS

The results of this prospective cohort study show that perinatal factors, which have previously been shown to be related to the development of the human microbiota, were not associated with the common infections found in infants, except for an increased occurrence of UTIs in those born via cesarean section. The present findings suggest that the burden of common respiratory infections in infants does not depend on the mode of delivery or intrapartum antibiotics, but is more closely related to other environmental factors.

## ACKNOWLEDGMENTS

We thank the nurses who worked with these children, especially Jaana Valkeapää, along with Heli Suomalainen, Anna Mantere, Eevi Heitto and Janica Bergström. We also thank Alise Ponsero and Evgenia Dikareva, MSc, for their contributions to the curation of the questionnaire data. Finally, we thank the participating families for their efforts.

## Supplementary Material



## References

[1] Gruber C Keil T Kulig M; MAS-90 Study Group. History of respiratory infections in the first 12 yr among children from a birth cohort. Pediatr Allergy Immunol. 2008;19:505–512. 18167154 10.1111/j.1399-3038.2007.00688.x

[2] Koopman LP Smit HA Heijnen ML. Respiratory infections in infants: interaction of parental allergy, child care, and siblings-- the PIAMA study. Pediatrics. 2001;108:943–948. 11581448 10.1542/peds.108.4.943

[3] Langer S Horn J Gottschick C. Symptom burden and factors associated with acute respiratory infections in the first two years of life-results from the LoewenKIDS cohort. Microorganisms. 2022;10:111. 35056559 10.3390/microorganisms10010111 PMC8781593

[4] Vissing NH Chawes BL Rasmussen MA. Epidemiology and risk factors of infection in early childhood. Pediatrics. 2018;141:06. 10.1542/peds.2017-0933 29794229

[5] Enserink R Lugner A Suijkerbuijk A. Gastrointestinal and respiratory illness in children that do and do not attend child day care centers: a cost-of-illness study. PLoS One. 2014;9:e104940. 25141226 10.1371/journal.pone.0104940 PMC4139325

[6] Fendrick AM Monto AS Nightengale B. The economic burden of non-influenza-related viral respiratory tract infection in the united states. Arch Intern Med. 2003;163:487–494. 12588210 10.1001/archinte.163.4.487

[7] Kusel MMH de Klerk N Holt PG. Occurrence and management of acute respiratory illnesses in early childhood. J Paediatr Child Health. 2007;43:139–146. 17316187 10.1111/j.1440-1754.2007.01033.x

[8] Latzin P Frey U Roiha HL; Swiss Paediatric Respiratory Research Group. Prospectively assessed incidence, severity, and determinants of respiratory symptoms in the first year of life. Pediatr Pulmonol. 2007;42:41–50. 17123315 10.1002/ppul.20542

[9] Forssell G Hakansson A Mansson NO. Risk factors for respiratory tract infections in children aged 2-5 years. Scand J Prim Health Care. 2001;19:122–125. 11482413 10.1080/028134301750235376

[10] von Linstow M Holst KK Larsen K. Acute respiratory symptoms and general illness during the first year of life: a population-based birth cohort study. Pediatr Pulmonol. 2008;43:584–593. 18435478 10.1002/ppul.20828

[11] Zutavern A Rzehak P Brockow I; LISA Study Group. Day care in relation to respiratory-tract and gastrointestinal infections in a German birth cohort study. Acta Paediatr. 2007;96:1494–1499. 17666100 10.1111/j.1651-2227.2007.00412.x

[12] Ball TM Castro-Rodriguez JA Griffith KA. Siblings, day-care attendance, and the risk of asthma and wheezing during childhood. N Engl J Med. 2000;343:538–543. 10954761 10.1056/NEJM200008243430803

[13] Jones LL Hashim A McKeever T. Parental and household smoking and the increased risk of bronchitis, bronchiolitis and other lower respiratory infections in infancy: systematic review and meta-analysis. Respir Res. 2011;12:5. 21219618 10.1186/1465-9921-12-5 PMC3022703

[14] Cushing AH Samet JM Lambert WE. Breastfeeding reduces risk of respiratory illness in infants. Am J Epidemiol. 1998;147:863–870. 9583717 10.1093/oxfordjournals.aje.a009540

[15] Toivonen L Karppinen S Schuez-Havupalo L. Longitudinal changes in early nasal microbiota and the risk of childhood asthma. Pediatrics. 2020;146:10. 10.1542/peds.2020-0421 32934151

[16] Toivonen L Schuez-Havupalo L Karppinen S. Antibiotic treatments during infancy, changes in nasal microbiota, and asthma development: population-based cohort study. Clin Infect Dis. 2021;72:1546–1554. 32170305 10.1093/cid/ciaa262 PMC8096219

[17] Mizutani T Aboagye SY Ishizaka A. Gut microbiota signature of pathogen-dependent dysbiosis in viral gastroenteritis. Sci Rep. 2021;11:13945. 34230563 10.1038/s41598-021-93345-y PMC8260788

[18] Paalanne N Husso A Salo J. Intestinal microbiome as a risk factor for urinary tract infections in children. Eur J Clin Microbiol Infect Dis. 2018;37:1881–1891. 30006660 10.1007/s10096-018-3322-7

[19] Hakim H Dallas R Wolf J. Gut microbiome composition predicts infection risk during chemotherapy in children with acute lymphoblastic leukemia. Clin Infect Dis. 2018;67:541–548. 29518185 10.1093/cid/ciy153 PMC6070042

[20] Jokela R Korpela K Jian C. Quantitative insights into effects of intrapartum antibiotics and birth mode on infant gut microbiota in relation to well-being during the first year of life. Gut Microbes. 2022;14:2095775. 36174236 10.1080/19490976.2022.2095775 PMC9542534

[21] Tapiainen T Koivusaari P Brinkac L. Impact of intrapartum and postnatal antibiotics on the gut microbiome and emergence of antimicrobial resistance in infants. Sci Rep. 2019;9:10635. 31337807 10.1038/s41598-019-46964-5 PMC6650395

[22] Korpela K Dikareva E Hanski E. Cohort profile: finnish health and early life microbiota (HELMi) longitudinal birth cohort. BMJ Open. 2019;9:e028500. 10.1136/bmjopen-2018-028500 PMC6609051 31253623

[23] Official Statistics of Finland (OSoF). Perinatal Statistics – Parturients, Deliveries and Newborns. National institute for health and welfare (THL). Available at: https://urn.fi/URN:NBN:fi-fe2018103146930. Accessed September 9, 2022.

[24] Backhed F Roswall J Peng Y. Dynamics and stabilization of the human gut microbiome during the first year of life. Cell Host Microbe. 2015;17:690–703. 25974306 10.1016/j.chom.2015.04.004

[25] Li W Tapiainen T Brinkac L. Vertical transmission of gut microbiome and antimicrobial resistance genes in infants exposed to antibiotics at birth. J Infect Dis. 2021;224:1236–1246. 32239170 10.1093/infdis/jiaa155 PMC8514186

[26] Magnus MC Haberg SE Stigum H. Delivery by Cesarean section and early childhood respiratory symptoms and disorders: the Norwegian mother and child cohort study. Am J Epidemiol. 2011;174:1275–1285. 22038100 10.1093/aje/kwr242 PMC3254156

[27] Poyry H Raappana A Kiviniemi M. Etiology of infectious diseases in acutely ill children at a pediatric hospital in Finland. Pediatr Infect Dis J. 2021;40:e245–e247. 33956758 10.1097/INF.0000000000003091 PMC8104009

[28] Moore RL Geraghty AA Feehily C. Can a probiotic supplement in pregnancy result in transfer to the neonatal gut: a systematic review. Acta Obstet Gynecol Scand. 2020;99:1269–1277. 32400910 10.1111/aogs.13899

[29] Leeder SR Corkhill R Irwig LM. Influence of family factors on the incidence of lower respiratory illness during the first year of life. Br J Prev Soc Med. 1976;30:203–212. 1009269 10.1136/jech.30.4.203 PMC478967

[30] Duijts L Jaddoe VWV Hofman A. Prolonged and exclusive breastfeeding reduces the risk of infectious diseases in infancy. Pediatrics. 2010;126:e18–e25. 20566605 10.1542/peds.2008-3256

[31] Frank NM Lynch KF Uusitalo U; TEDDY Study Group. The relationship between breastfeeding and reported respiratory and gastrointestinal infection rates in young children. BMC Pediatr. 2019;19:339. 31533753 10.1186/s12887-019-1693-2 PMC6749679

[32] Lopez-Alarcon M Villalpando S Fajardo A. Breast-feeding lowers the frequency and duration of acute respiratory infection and diarrhea in infants under six months of age. J Nutr. 1997;127:436–443. 9082027 10.1093/jn/127.3.436

[33] Bergroth E Remes S Pekkanen J. Respiratory tract illnesses during the first year of life: effect of dog and cat contacts. Pediatrics. 2012;130:211–220. 22778307 10.1542/peds.2011-2825

[34] Hatakka K Piirainen L Pohjavuori S. Factors associated with acute respiratory illness in day care children. Scand J Infect Dis. 2010;42:704–711. 20465487 10.3109/00365548.2010.483476

[35] Matharu D Ponsero AJ Dikareva E. Bacteroides abundance drives birth mode dependent infant gut microbiota developmental trajectories. Front Microbiol. 2022;13:953475. 36274732 10.3389/fmicb.2022.953475 PMC9583133

