# Supplementary material for: Perinatal and Other Risk Factors for Common Infections in Infancy: A Prospective Cohort Study
Source: Pediatr Infect Dis J. 2023 Sep 14;42(12):e447–53. doi: 10.1097/INF.0000000000004112 (PMC10629602; doi:10.1097/INF.0000000000004112)
Supplement: Supplementary file 1 [file inf-42-e447-s001.docx]

| **Supplemental Digital Content 1.** Definitions of infectious diseases episodes used in the statistical analysis. | | |
| --- | --- | --- |
| Infection | Diagnosis made  at Home (H)  at Health Care (HC) | Reported symptoms or diagnoses |
| Respiratory Tract Infections | H + HC | Upper + Lower Respiratory Tract Infections |
| Upper Respiratory Tract Infections | H + HC | Common cold, cough, laryngitis, influenza, otitis media, tonsillitis, or pharyngitis |
| Lower Respiratory Tract Infections | HC | Pneumonia, viral wheeze, or bronchiolitis |
| Gastrointestinal Infection | H + HC | Diarrhea and vomiting together or separately. Fever included when reported together with only gastrointestinal symptoms. |
| Urinary Tract Infections | HC | Pyelonephritis or urinary tract infection |
| Other Infections | H + HC | Isolated fever, fever and skin lesions, other viral infection, staphylococcal skin infection, balanitis, skin infections, osteomyelitis, cellulitis or sepsis |
